# Self-Assembly Behavior and pH-Stimuli-Responsive Property of POSS-Based Amphiphilic Block Copolymers in Solution

**DOI:** 10.3390/mi9060258

**Published:** 2018-05-24

**Authors:** Yiting Xu, Kaiwei He, Hongchao Wang, Meng Li, Tong Shen, Xinyu Liu, Conghui Yuan, Lizong Dai

**Affiliations:** Fujian Provincial Key Laboratory of Fire Retardant Materials, College of Materials, Xiamen University, Xiamen 361005, China; hekaiwei@stu.xmu.edu.cn (K.H.); 20720171150035@stu.xmu.edu.cn (H.W.); 20720171150098@stu.xmu.edu.cn (M.L.); 20720171150102@stu.xmu.edu.cn (T.S.); lxy212@xmu.edu.cn (X.L.); yuanch@xmu.edu.cn (C.Y.)

**Keywords:** stimuli-responsive polymer, polyhedral oligomeric silsesquioxane, self-assembly, protein adsorption resistance, controlled release

## Abstract

Stimuli-responsive polymeric systems containing special responsive moieties can undergo alteration of chemical structures and physical properties in response to external stimulus. We synthesized a hybrid amphiphilic block copolymer containing methoxy polyethylene glycol (MePEG), methacrylate isobutyl polyhedral oligomeric silsesquioxane (MAPOSS) and 2-(diisopropylamino)ethyl methacrylate (DPA) named MePEG-*b*-P(MAPOSS-*co*-DPA) via atom transfer radical polymerization (ATRP). Spherical micelles with a core-shell structure were obtained by a self-assembly process based on MePEG-*b*-P(MAPOSS-*co*-DPA), which showed a pH-responsive property. The influence of hydrophobic chain length on the self-assembly behavior was also studied. The pyrene release properties of micelles and their ability of antifouling were further studied.

## 1. Introduction

Recently, stimuli-responsive polymers have attracted great attention because of their unique properties. The characteristics of these smart polymers can be adjusted by temperature, light, pH values, ionic strength, enzyme proteins, antigens, and so on [1,2,3,4,5,6]. Among stimuli-sensitive copolymers, temperature- or pH-sensitive copolymers, mainly consisting of 2-diisopropylaminoethyl methacrylate (DPA) [7], 2-(dimethylamino)ethyl methacrylate (DMAEMA) [8,9,10] and *N*-isopropylacryl amide (NIPAM) [11] blocks, have been extensively investigated for their potential application in targeted drug delivery.

Block copolymers are composed of two or more chemical components connected by chemical bonds. The structure of block copolymer micelles is easily regulated by changing the environmental conditions, so the self-assembly of block copolymers has attracted wide interest in the field of self-assembly. Amphiphilic block copolymers spontaneously self-assemble into well-organized nanostructures in selective solvents. Depending on many factors such as water content, copolymer concentration and architecture of the amphiphilic macromolecules, the self-assembled structures could be of various morphologies, ranging from star-like spherical micelles to multicompartment nanostructures [12,13,14,15,16,17,18].

Polyhedral oligomeric silsesquioxane (POSS), classified as a unique inorganic silica nanoparticle with a uniform cubic structure, has been widely incorporated into polymer matrices to produce novel organic/inorganic hybrid materials. The POSS-based polymer acts as a hybrid material with excellent mechanical and inorganic properties. POSS with inert vertex groups on the silicon-oxygen cage renders it quite hydrophobic, therefore promoting the formation of a well-patterned micelles structure to control drug release [19,20,21]. The aggregation of POSS moieties, which are nanoscale at about 1.5 nm, can form a regular and stable hydrophobic core in micelles. The characteristic structure makes the encapsulating of hydrophobic drugs in micelles more advantageous. There is increasing interest in the synthesis, self-assembly and applications of stimuli-responsive POSS-based amphiphilic macromolecules. Recent advances in the field of synthetic polymer chemistry, especially in controlled radical polymerizations, such as ATRP and various click reactions, have paved the way for the introduction of POSS into well-defined copolymer architectures. Matyjaszewski et al. were the first to report POSS containing well-defined P(MA-POSS)-*b*-poly(*n*-butyl acrylate)-*b*-P(MA-POSS) triblock copolymers via ATRP, and their self-assembly behaviors in films [22]. Deng et al. prepared block copolymers of MA-POSS and PMMA by reversible addition-fragmentation chain transfer (RAFT) polymerization and studied their self-assembly to gain nanostructured hybrid polymer networks [23]. Cheng et al. prepared an amphiphilic copolymer, polystyrene-(carboxylic acid-functionalized POSS) (PS-APOSS), and the assembled structure including vesicles, worm-like cylinders and spheres was obtained by changing the ionization degree of the carboxylic acid [24]. He et al. synthesized amphiphilic POSS-containing copolymers poly(acrylic acid)-*co*-poly(acrylate-POSS) via ATRP, and the self-assembled structure could be tuned in aqueous solution by regulating the molar ratio of poly(acrylic acid) to poly(acrylate-POSS) in the copolymer [25]. This exciting discovery provides a totally wide and novel window for designing amphiphilic polymers due to ATRP being a simple and powerful method to synthesize diverse polymers.

**Scheme 1:**
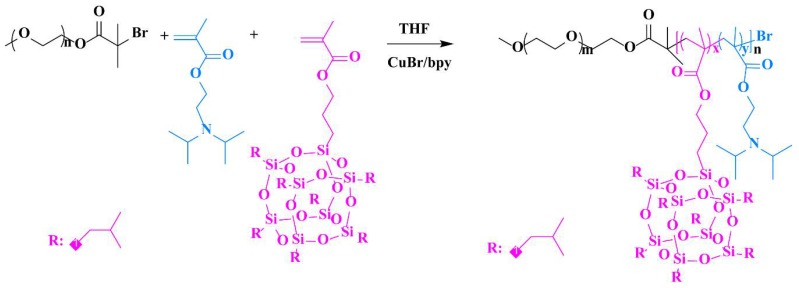
Synthetic way of MePEG-*b*-P(MAPOSS-*co*-DPA).

In this work, a novel hybrid amphiphilic block copolymer was prepared with MePEG as the backbone connecting another block with MAPOSS and DPA units via ATRP. The synthetic route is described in Scheme 1. Owning to the unique amphiphilic architecture, MePEG-*b*-P(MAPOSS-*co*-DPA) could form micelles. The self-assembly and pH-sensitive behaviors of copolymers in aqueous solution were studied by dynamic light scattering (DLS) and transmission electron microscopy (TEM), including the cladding and release of pyrene. Meanwhile, the antifouling ability of the assembly was systematically studied.

## 2. Experimental Section

### 2.1. Materials

2-(Diisopropylamino)ethyl methacrylate (DPA) (97%, Aladdin Co., Shanghai, China) was purified by basic alumina columns prior to use. Methoxy polyethylene glycol (MePEG 5000) was freeze-dried for 24 h to remove water. Methacrylate isobutyl POSS (MAPOSS) purchased from Hybrid Plastic Co. Styrene (Sinoreagent Co., Shanghai, China) was purified by passing through a column filled with basic Al_2_O_3_ prior to use. Other chemicals were purchased from Sinoreagent Co. and used as received.

### 2.2. Synthesis of MePEG-Br Macroinitiator

The MePEG-Br was synthesized using the methods described in the literature [26]. Typically, the methoxy polyethylene glycol (MePEG) (5.0 g, 1.0 mmol) and triethylamine (TEA) (2.2 g, 4.0 mmol) were dissolved in dichloromethane (DCM) (20.0 mL) and cooled with an ice-salt bath below 0 °C. Then, bromoisobutyryl bromide (BiBB) (0.368 g, 3.2 mmol) dissolved in DCM (2.0 mL) was slowly added dropwise to the reaction solution. The solution was slowly warmed to room temperature and reacted for 24 h. The product was dissolved in distilled water and DCM removed by rotary evaporation. Finally, the macroinitiator MePEG-Br was obtained as a white powder. The product was characterized by ^1^H-NMR spectroscopy (Figure S1).

^1^H-NMR (CDCl_3_, ppm, TMS) d: 3.39 ppm [3H, CH_3_-O-], 3.66 ppm [2H, -(OCH_2_)n-], 4.34 ppm [2H, -(OCH_2_)n-CH_2_O-], 1.95 ppm [6H, -OCOC(CH_3_)_2_Br].

### 2.3. Synthesis of MePEG-b-P(MAPOSS-co-DPA)

A series of amphiphilic copolymers MePEG-*b*-P(MAPOSS-*co*-DPA) via ATRP was synthesized. MePEG-Br (2.96 g, 0.5 mmol), DPA (0.15 g, 0.03 mol), MAPOSS (5.11 g, 5.5 mmol), 2-dipyridyl (bpy) (0.15 g, 1 mmol) and anhydrous 1,4-dioxane (2.0 mL) were added into a 10-mL Schlenk tube, followed by three freeze-pump-thaw cycles. Then, CuBr (0.07 g, 0.5 mmol) was added into the Schlenk tube under the protection of argon, followed by a freeze-pump-thaw cycle. Next, this Schlenk tube was heated to 70 °C, and polymerization was performed for 24 h under continuous stirring. Then, the reaction solution was passed through the Al_2_O_3_ column for the removal of the catalyst. Finally, the solvent was removed by rotary evaporation, and the product was dried until constant weight in a vacuum oven at 40 °C. The preparation of MePEG-*b*-P(MAPOSS-*co*-DPA) with different contents of MAPOSS was then performed in a similar way.

### 2.4. Self-Assembly Procedure of MePEG-b-P (MAPOSS-co-DPA) Amphiphilic Copolymer

A series of micellar solutions was prepared by solution volatilization-induced self-assembly [27]. The POSS-based amphiphilic copolymer MePEG-*b*-P(MAPOSS-*co*-DPA) was dissolved in tetrahydrofuran (THF) to get the polymer mother liquor. Then, the liquid was added dropwise to a certain volume of ultrapure water, and the mixture was stirred at ambient temperature until the THF was completely volatilized to get the amphiphilic copolymer micelles. Typical self-assembly solutions were prepared as follows:

MePEG-*b*-P(MAPOSS-*co*-DPA) (5 mg) was dissolved in 1 mL of THF. Then, the solution was gradually diluted by 5 mL of deionized water. The solution was stirred overnight to completely remove THF at room temperature. The final solution of self-assembled micelles was used for the following characterizations. A series of different concentrations of hydrochloric acid and sodium hydroxide was selected to adjust the pH of the micellar solution.

### 2.5. Preparation of Pyrene Encapsulated Micelles

Zero-point-one milliliters of pyrene in acetone (10 mg/mL) were added to the serum vial, and acetone was evaporated under continuous flow of argon. Then, 25 mL of polymer micelle solution (1 mg/mL) were added and sonicated for 1 h to ensure encapsulation of the pyrene. Finally, after the loaded pyrene was removed by filtration, a pyrene-loaded micelle solution was successfully prepared.

### 2.6. Preparation of Micelles and Fetal Bovine Serum Mixed Solution for Anti-Protein Adsorption Performance Test

The amphiphilic copolymer MePEG-*b*-P(MAPOSS-*co*-DPA) was dissolved in THF, and the resulting polymer mother liquor was slowly added dropwise to the fetal bovine serum (FBS) solution until the THF was completely volatilized. Then, the mixed solution was obtained.

### 2.7. Characterization

The ^1^H-NMR measurements were carried out on a Bruker AV400 MHz NMR spectrometer (Bruker, Geneva, Switzerland) at room temperature with tetramethylsilane (TMS) as the internal standard and CDCl_3_ as a solvent. Fourier-transform infrared spectrometry (FTIR) spectra were recorded on Nicolet Avatar 360 FTIR (Thermo Fisher Scientific, Shanghai, China). The molecular weight was measured by gel permeation chromatography (GPC) on the APC Installation Kit. Data were obtained using the PSS WinGPC system. THF was used as the eluent at a flow rate of 0.5 mL/min. A series of low polydispersity polystyrene standards were used for the GPC calibration.

The optical transmittance of the MePEG-*b*-P(MAPOSS-*co*-DPA) solution was obtained on a UV-2550 spectrometer (SHIMADZU, Kyoto, Japan) at 500 nm. Transmission electron microscopy (TEM) images were conducted using a JEM2100 transmission electron microscope (JEOL, Tokyo, Japan) with an accelerating voltage of 200 kV. One drop of micelles solution was placed on a copper-mesh coated with carbon and then air-dried before measurement. Zeta potential measurements and dynamic light scattering (DLS) were recorded on a Zetasizer NanoZS Instrument (Malvern Instruments, Malvern, UK) at a wavelength of 500 nm. Fluorescence spectroscopy was conducted on the F7000 fluorescence spectrophotometer (Hitachi, Tokyo, Japan). Both the excitation and emission slit widths were 2.5 nm, and the scan rate was 240 nm/min.

## 3. Results and Discussion

### 3.1. Synthesis of MePEG-b-P(MAPOSS-co-DPA)

The macroinitiator MePEG-Br was used to initiate the ATRP polymerization of MAPOSS and DPA monomers. In this paper, three kinds of amphiphilic copolymers with different contents of MAPOSS segments were designed and synthesized, named as BCP1, BCP2, BCP3, respectively. Experimental conditions and results of amphiphilic polymer with MAPOSS and DPA are shown in Table 1.

^1^H-NMR spectra of MePEG-*b*-P(MAPOSS-*co*-DPA) with various molecular weight are shown in Figure S2. The characterization analysis of BCP2 was used as an example. The MAPOSS structural unit has one characteristic signal at 0.61 ppm (Peak b and Peak c). The new signals at 2.65 ppm (Peak d) and 3.02 ppm (Peak e) correspond to hydrogen in methylene (-NCH_2_-) and methine (-NCH-), respectively, which are attached to tertiary amine groups in the DPA structure. The integral ratio of Peak d and Peak e is 1:1, which is consistent with the theoretical ratio. The methylene peak attached to the MePEG backbone (-CH_2_-COO-) appears at 3.66 ppm (Peak a). What is more, there is no characteristic double bond peak appearing at 5.5–6.5 ppm, indicating that there was no residual monomer. This indicates that the two monomers were successfully introduced into the polymer molecular chain.

FTIR (Figure S3) was used to further certify the successful synthesis of the hybrid POSS-based copolymers. Peaks at 1096 cm^−1^ are attributed to asymmetric vibrational stretching of the cage-shaped skeleton, indicating that MAPOSS has been successfully introduced into the copolymer. The characteristic peaks at 2865 cm^−1^ and 972 cm^−1^ correspond to C-H stretching vibration on -NCH- and C-N vibration, respectively, implying the successful synthesis of the copolymer. The C=O stretching vibration of MAPOSS and DPA occurs at 1725 cm^−1^. GPC analysis of the samples also exhibited that the molecular weight is relatively uniform with well-defined and relatively narrow polydispersity (Figure S4). The GPC curve of each copolymer has a certain tail, mainly due to the high content of MePEG structural units in MePEG-*b*-P(MAPOSS-*co*-DPA). The -C-O-C- structure in the main chain makes the copolymer easily adsorbed by the stationary phase of the ultra-high-efficiency polymer column, resulting in the increased leaching time and trailing of the GPC curve.

### 3.2. Self-Assembly Behavior of MePEG-b-P(MAPOSS-co-DPA) in Aqueous Solution

**Figure 1:**
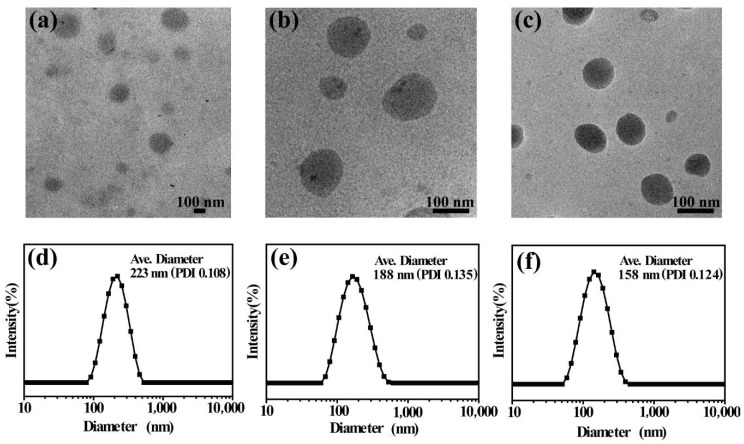
TEM images and DLS curves of BCP1 (**a**,**d**), BCP2 (**b**,**e**) and BCP3 (**c**,**f**) micelles.

To explore the influence of the hydrophobic ratio on the self-assembly system, we chose three amphiphilic copolymers (BCP1, BCP2 and BCP3) with the same MePEG units and different hydrophobic content. The segment lengths of MePEG and DPA structural units are the same, while the proportion of the MAPOSS segment is different. The TEM photos of the copolymer micelles and the results of the DLS test are shown in Figure 1. From the DLS results, it can be seen that the micelle size of BCP1 is 223 nm. As the proportion of the hydrophobic segment gradually increases, the micelle size of BCP2 and BCP3 decreased to 188 nm and 158 nm, respectively. The hydrodynamic diameter of the micelle measured by DLS is usually larger than the micelle observed by TEM, because DLS measures swollen micelles in aqueous solutions, and the sizes observed by TEM are derived from the dried micelles.

The self-assembly behavior of MePEG-*b*-P(MAPOSS-*co*-DPA) in aqueous solution was investigated. With the increase of the MAPOSS unit number, the nanometer size of micelles decreases in sequence. This is because the MAPOSS has a strong hydrophobicity, and its increased content can significantly enhance the hydrophobicity of the copolymer chain in solution. Therefore, the copolymer is driven by a relatively larger hydrophobic effect in the process of forming a gel, resulting in a smaller micelle size. The result is consistent with the report of Zhang et al. [28].

The critical micelle concentrations (CMC) of BCP1, BCP2 and BCP3 are also measured by a fluorescence method with hydrophobic pyrene as a fluorescent probe. Fluorescence spectroscopy is utilized to monitor the self-assembly process (Figure S5). The intensity ratio (*I*_1_/*I*_3_) of the peaks located at 373 nm and 383 nm from the pyrene emission spectra can be taken into account to know the local environment of pyrene. A lower value of *I*_1_/*I*_3_ indicates that pyrene is located in the hydrophobic environment. From the concentration variable experiment, the *I*_1_/*I*_3_ values reflect the CMC value [29,30]. The CMC of BCP1, BCP2 and BCP3 is found to been 0.016 mg/mL, 0.015 mg/mL and 0.012 mg/mL, respectively. Despite the three units ratio being pretty similar in BCP1, BCP2 and BCP3, the small increase of POSS content improved the hydrophobicity of the whole polymer chain, resulting in the lowest CMC of BCP3. Furthermore, the longer polymer chain length possesses a stronger intermolecular interaction. These factors can increase the driving force of the self-assembly process, which means that this amphiphilic copolymer can form micelles at a lower concentration.

**Figure 2:**
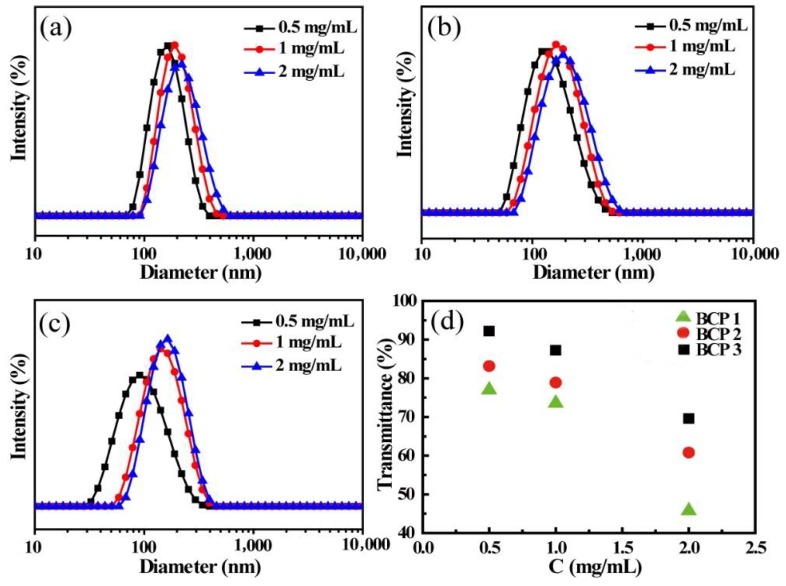
DLS curves of BCP1 (**a**), BCP2 (**b**) and BCP3 (**c**) micelles at various initial concentration of 0.5 mg/mL, 1 mg/mL and 2 mg/mL; (**d**) optical transmittance at 500 nm with different concentration for BCP1, BCP2 and BCP3.

In order to explore the effect of initial concentration on the self-assembly of MePEG-*b*-P(MAPOSS-*co*-DPA) solution, the micellar size of amphiphilic block copolymers with initial concentrations was investigated, and the results are shown in Figure 2. It is found that the size of micelles increase with the increasing initial concentrations, indicating that the size of micelles was significantly affected by the initial concentration of the amphiphilic copolymer solution. Ultraviolet transmittance is a common method to assist in the characterization of micelle size in solution. When the micelle size of the same sample increases with the increasing concentration, the transmittance to ultraviolet light of specific wavelength decreases. The ultraviolet transmittance of BCP1, BCP2 and BCP3 micelles at different initial concentrations is shown in Figure 2d. As the initial concentration of the amphiphilic copolymer solution increases, the corresponding UV transmittance becomes smaller. The change of UV transmittance with different initial concentrations is consistent with the DLS results. From this phenomenon, we can conclude that the initial concentration of the amphiphilic copolymer solution affects the micellar size. Copolymer micelles with higher initial concentrations favor larger micellar size.

### 3.3. pH-Responsive Behavior of MePEG-b-P (MAPOSS-co-DPA) Copolymers

**Figure 3:**
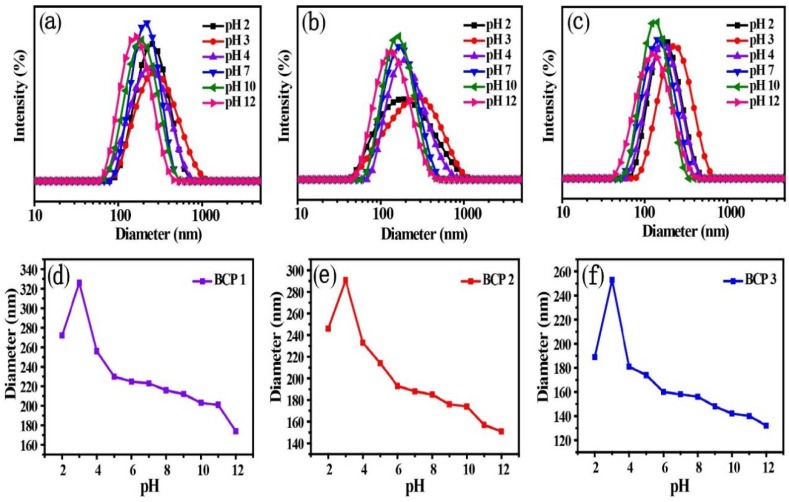
DLS plots of amphiphilic copolymer aggregates and schematic of the aggregate size variation of BCP1 (**a**,**d**), BCP2 (**b**,**e**) and BCP3 (**c**,**f**) under different pH values of aqueous solution.

DPA is typically used as a monomer for the preparation of pH-responsive polymers for changing the hydrophilicity under different pH conditions. The DLS curves and micelle size distribution of the amphiphilic copolymer micelles under different pH values are shown in Figure 3. It shows that the micelle size changes with the pH value of the aqueous solution, and the samples with a different proportion of MAPOSS segments have a similar tendency of diameter variations. That is, with the decreasing of pH values in an acid solution system, the size of the micelles of the copolymers increases gradually. Once the pH decreases from 3–2, the micelles slightly decrease. On the other hand, when the pH of the solution increases from 3–12, the micelle size decreases gradually.

In an acid solution system, the protonation of the tertiary amine group in DPA units is high, which make DPA become hydrophilic and diffuse to the shell. There is a strong electrostatic repulsive force between DPA units, leading to DPA units’ greater attraction to water molecules. Therefore the hydrodynamic size of the micelles increases in the acidic system. By further decreasing the pH, the effect can become stronger. However, DPA segments become hydrophobic due to deprotonation in alkaline conditions, which induces the micelles to shrink. The hydrodynamic size of micelles decreases with increasing pH value.

**Figure 4:**
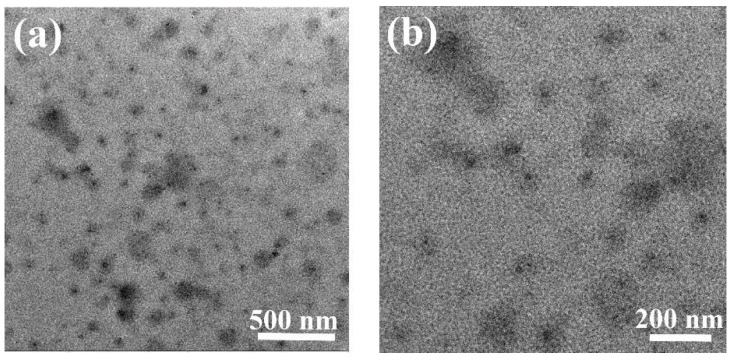
TEM image of micelles in aqueous solution with pH = 2: (**a**) lower magnification and (**b**) higher magnification.

Once the pH dropped to two, a strong acidic system causes some of the POSS cages to fall off and the micelles to disassemble, resulting in a smaller average micelle size. This is because of the hydrolysis of ester groups of POSS segments on the polymer chain [31]. POSS has a deep contrast under the observation of TEM, so black spots can be observed in Figure 4.

**Figure 5:**
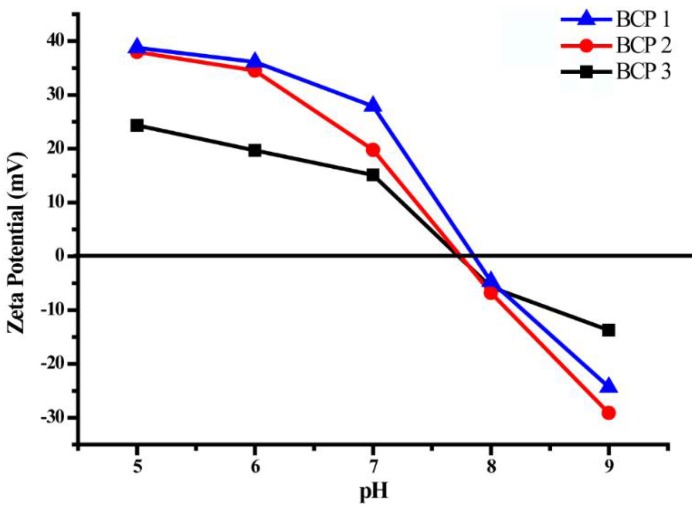
Zeta potential of amphiphilic block copolymers aggregates BCP1, BCP2 and BCP3.

The surface zeta potential of amphiphilic copolymers MePEG-*b*-P(MAPOSS-*co*-DPA) micelles at different pH values was determinated by Zetasizer NanoZS Instrument. As shown in Figure 5, the zeta potential on the micelle surface is positive in acidic systems, as well as negative in alkaline systems. The isoelectric point (pI) values of BCP1, BCP2 and BCP3 micelles are 7.85, 7.74 and 7.71. It can be seen that with the increase of hydrophobic MAPOSS structure units, the relative proportion of DPA to MAPOSS units is 4.72, 4.48 and 4.16, thus resulting in a lower pI value. Under acidic conditions, the zeta potential on the micelle surface is positive due to the protonation of DPA structure units. However, protonated DPA segments become deprotonated under alkaline condition, resulting in the negative zeta potential on the micelle surface.

### 3.4. Controlled Release of Pyrene from Amphiphilic Block Copolymers Micelles

**Figure 6:**
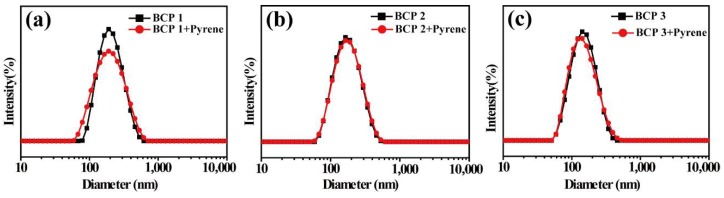
DLS curves of (**a**) BCP1, (**b**) BCP2 and (**c**) BCP3 micelles before and after encapsulating pyrene.

Drug release is an important application for responsive polymer, and the preparation of fluorescent molecules is the primary means to evaluate drug release efficiency [32,33,34,35]. Herein, the release of pyrene as a drug model from the micelles by adjusting the pH value is investigated. During the self-assembly process of copolymer micelles, pyrene is added in the aqueous solution. As can be seen from Figure 6, the size of the micelles encapsulating pyrene does not change obviously, and the PDI increased slightly. The self-assembled copolymer BCP2 formed in aqueous solution has a micelle size of 188 nm and a PDI of 0.135. When the micelles are loaded with fluorene molecules, the particle size is 191.8 nm, and the PDI is 0.153. Similarly, the sizes of BCP1 and BCP3 micelles do not change much before and after encapsulation. It is indicated that the process of loading the hydrophobic molecules does not affect the self-assembly process of the polymer.

**Figure 7:**
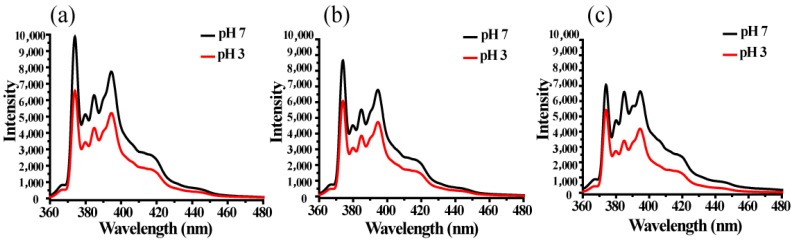
The fluorescent spectra of the micellar solution under different pH values: (**a**) BCP1; (**b**) BCP2; (**c**) BCP3.

The conditions of pH = 3 and pH = 7 were selected to study the encapsulation behavior of micelles on pyrene. It can be seen from Figure 7 that when the pH is seven, the fluorescence intensity of BCP1 micelles encapsulated with pyrene is the highest, and the intensity of BCP2 and BCP3 decreases sequentially. The result is reasonable because the micelle size of BCP3 is the smallest, resulting in less encapsulated pyrene molecules; therefore, the corresponding fluorescence intensity decreases. When the pH is at seven, MePEG-*b*-P(MAPOSS-*co*-DPA) micelles contain more fluorescent molecules and thus have higher fluorescence intensity. Once the pH decreased from 7–3, the micelles loaded less fluorescent molecules and had lower fluorescence intensity.

From the above results, we can conclude that the micelle had a relatively compact core because of the low protonation of DPA when the pH was seven near the isoelectric point of the micelles. This is favorable for micelle core loading of guest molecules stably. However, under acidic conditions (pH = 3), the tertiary amine groups of the DPA segment are protonated. The micelles are in a swelling state in the aqueous solution, which facilitates the release of the pyrene.

**Figure 8:**
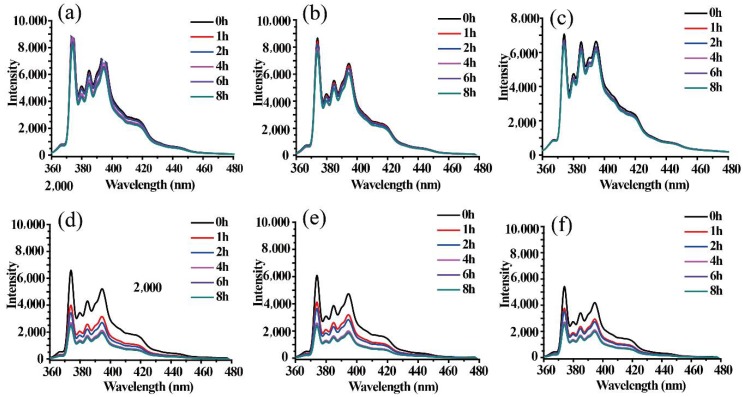
The fluorescent emission spectra of BCP1, BCP2 and BCP3 micelles encapsulated with pyrene in aqueous solution when pH = 7 (**a**–**c**) and pH = 3 (**d**–**f**) under an excitation wavelength of 336 nm along time.

The fluorescence intensity of MePEG-*b*-P(MAPOSS-*co*-DPA) micelles loaded with pyrene in different pH varies with time as shown in Figure 8. In order to reflect the effect of acid conditions on the release rate and degree of pyrene micelles, the release of pyrene from BCP1, BCP2 and BCP3 micelles with different contents of MAPOSS is determined by recording the fluorescence spectra with respect to time, as shown in Figure S6. In the fluorescence spectra of pyrene, the peak intensity of *I*_1_ and *I*_3_ represents the hydrophilic environment and the hydrophobic environment, respectively [36]. Therefore, the cumulative fraction of released pyrene can be estimated from the ratio of (*I*_30_ − *I*_3_)/*I*_30_. *I*_30_ and *I*_3_ represent the initial fluorescence intensity and fluorescence intensity at a certain time, respectively. It is easy to find that when the pH is seven, the fluorescence intensity of BCP1, BCP2 and BCP3 decreases slightly within 8 h, indicating that the micelles can load pyrene stably. When the pH drops to three, the fluorescence intensity decreases with time. Notably, the fluorescence intensity decreases faster within 1 h from the beginning. In other words, polymer micelles BCP1 released the most pyrene molecules within 8 h, followed by BCP2 micelles and BCP3 micelles in an acidic environment, and their cumulative release percentages were decreased, which were 62.33%, 61.07% and 52.79% respectively.

**Figure 9:**
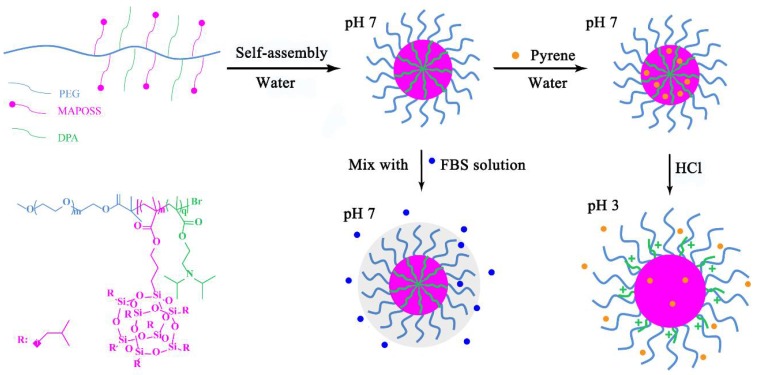
The schematic illustration of the self-assembly of MePEG-*b*-P(MAPOSS-*co*-DPA) micelles.

In conclusion, the proportion of MAPOSS structural units increases sequentially in BCP1, BCP2 and BCP3. The hydrophobicity of the micelles also increases; the aggregation in the micelle core is more compact; and the fluorescent molecules are more difficult to release. However, tertiary amine groups of DPA units are positively charged under acidic conditions and become hydrophilic, which induce DPA to diffuse to the shell. The electrostatic repulsion of DPA units results in a large degree of looseness in the micelle. Pyrene molecules are easily released through the loose shell. Based on the above results, we present the mechanism diagram of the release of pyrene from MePEG-*b*-P(MAPOSS-*co*-DPA) micelles. As shown in Figure 9, the encapsulation and release of the fluorescent molecules are controlled by the loosening or compaction of the micelles. When the pH is seven (near the equipotential point of micelle), there is a compact micelle because of the low protonation of DPA. This is conducive to the stabilization of pyrene loaded in the micellar cores. However, DPA segments in hydrophobic blocks are protonated under acidic conditions and diffuse to the shell. The same type of charge repulsion occurs between the positively-charged DPA segments, and the molecular chain is more stretched. The micelles are in a swollen state in the aqueous solution, which facilitates the release of the pyrene in the micelles.

### 3.5. Anti-Protein Adsorption Properties of MePEG-b-P(MAPOSS-co-DPA) Micelles

**Figure 10:**
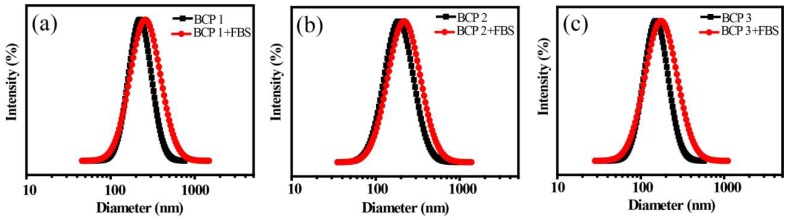
DLS results of micelles before and after mixing with FBS: (**a**) BCP1; (**b**) BCP2; (**c**) BCP3.

The amphiphilic copolymers MePEG-*b*-P(MAPOSS-*co*-DPA) contained MePEG segments, which have a certain effect on the anti-protein adsorption ability of the system [37,38,39,40,41,42,43]. The anti-protein adsorption properties of the copolymer are investigated by mixing FBS solution with copolymers’ micellar solution. The DLS results of the copolymers with different content of MAPOSS segments with FBS at pH = 7 are shown in Figure 10. The particle sizes of BCP1, BCP2 and BCP3 micelles are all increased, but to a very small extent, and the PDI is also slightly increased. The results showed that the micelles could effectively inhibit the further adsorption of protein, and the micelles had a certain ability to resist protein adsorption.

**Figure 11:**
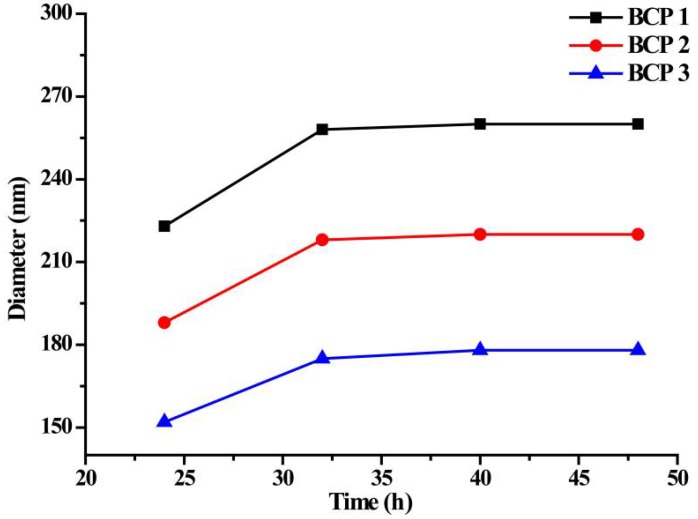
Hydrodynamic size of micelles versus time in FBS solution.

The ability of the amphiphilic copolymer micelles to resist proteins was mainly provided by the MePEG; however, DPA is often used as a protein adsorbent because of its ability to adsorb proteins. Therefore, there is a competitive relationship between the adsorption capacity of MePEG against protein and the protein adsorption capacity of DPA in the amphiphilic copolymer. The hydrodynamic size variation of micelles versus time in FBS solution is shown in Figure 11. As we can see, a slight increase of the hydrodynamic sizes of BCP1, BCP2 and BCP3 after mixing with FBS occurs within 32 h, and then, the micelle sizes keep stable. That is, when the adsorption equilibrium of the protein is reached, the three micellar sizes increased only by 20~30 nm compared to that before the adsorption of protein, indicating a certain ability of the micelle to resist protein. From the above results, we can conclude that MePEG segments in the micelles act as a shell layer; MAPOSS and DPA units are hydrophobic as the core during the formation of micelles in near-neutral media. At the same time, the molecular weight of the PEG used in the system is 5000, which is much larger than that of DPA content. Although the hydrophobic DPA proportion of the core is decreased gradually in the BCP1, BCP2 and BCP3 micelles, the ability of the anti-protein is mainly dominated by the MePEG content of the shell. It is calculated that the three copolymer micelles are coated by a long hydrophilic MePEG shell, suggesting the ability of the micelles to resist protein change a little.

Therefore, we believe that the interaction between the MePEG segment of amphiphilic copolymers and water molecule effectively inhibits the further adsorption of protein by micelles, which results in a slow increase of the micelle size, and micelles have anti-protein adsorption properties. The schematic of the mixing of micelles and FBS solution is also shown in Figure 9. Amphiphilic copolymers containing MePEG segments act as an anti-protein adsorption material, and its anti-protein adsorption mechanism is related to hydration. The combined water on the surface of the material prevents the material from adsorbing protein. The amphiphilic copolymers first combine a large number of water molecules around it by hydrogen bonding, forming a “hydration layer” of physical and energy barriers [44], preventing the micelles from adsorbing protein.

## 4. Conclusions

A hybrid amphiphilic block copolymer containing MAPOSS, MePEG and DPA via ATRP polymerization was synthesized. The results of ^1^H-NMR, FTIR and gel permeation chromatography indicated the well-defined structures of the copolymers. The copolymer has the ability to self-assemble into a spherical micelle in response to pH. With the increase of the pH value from 2–12, the size of micelle increases first, then decreases, indicating an outstanding pH-sensitive response. At the same pH, the more MAPOSS units, the tighter the micelle and the smaller the particle size of the micelle. With the fluorescent molecule pyrene as a hydrophobic drug model, MePEG-*b*-P(MAPOSS-*co*-DPA) can realize controlled release of fluorescent molecular pyrene in an acidic environment. The MAPOSS unit content affects the release percentage and release rate of pyrene. With the content of the MAPOSS units increasing, the degree of release of the fluorescent molecule pyrene from the micelle becomes smaller and the release rate becomes slower. The BCP3 micelles in an acidic environment show the smallest cumulative release percentages (52.79%). This is important for polymers with anti-protein adsorption ability to prevent biological pollution, which eventually averts the host immune response. The MePEG-*b*-P(MAPOSS-*co*-DPA) micelles containing MePEG segments effectively inhibit the micelles from adsorbing proteins and have good anti-protein adsorption ability.

## Figures and Tables

**Table 1:** Experiments and results of ATRP polymerization of MePEG-Br, MAPOSS and DPA.

Sample Name	[MePEG-Br]:[DPA]: [MAPOSS] ^a^	[MePEG-Br]:[DPA]: [MAPOSS] ^b^	Time (h)	$\bar{Mn}$ (^1^H-NMR)	$\bar{Mn}$ (GPC)	PDI
BCP1	1:60:11	1:50.06:10.60	24	25,839	26,314	1.09
BCP2	1:60:13	1:53.84:12.02	24	28,010	30,681	1.27
BCP3	1:60:15	1:57.66:13.86	24	30,566	35,908	1.23

^a^ The feed molar ratio of monomers; ^b^ the composition ratio in copolymer determined by ^1^H-NMR spectra.

## Supplementary Materials

The following are available online at http://www.mdpi.com/2072-666X/9/6/258/s1, Figure S1: ^1^H-NMR spectrum of the MePEG-Br macroinitiator, Figure S2: ^1^H-NMR spectra of MePEG-*b*-P(MAPOSS-*co*-DPA) with various molecular weights, Figure S3: FTIR spectrum of MePEG-*b*-P (MAPOSS-*co*-DPA), Figure S4: GPC traces of MePEG-*b*-P(MAPOSS-*co*-DPA), Figure S5: Fluorescence-emission spectrogram of pyrene in an aqueous solution of amphiphilic block copolymer: (a) BCP1; (c) BCP2 and (e) BCP3 with different concentrations (mg/mL); relationship between *I*_1_*/I*_3_ of pyrene and amphiphilic block copolymer concentration: (b) BCP1, (d) BCP2 and (f) BCP3, Figure S6: Cumulative pyrene release percentage of pyrene encapsulated by micelles of BCP1, BCP2 and BCP3 when pH = 7 and pH = 3.
