# Supplementary material for: Self-Assembly Behavior and pH-Stimuli-Responsive Property of POSS-Based Amphiphilic Block Copolymers in Solution
Source: Micromachines (Basel). 2018 May 24;9(6):258. doi: 10.3390/mi9060258 (PMC6187445; doi:10.3390/mi9060258)
Supplement: Supplementary file 1 [file micromachines-09-00258-s001.zip › micromachines-305128-SI.docx]

**Supplementary Materials:**


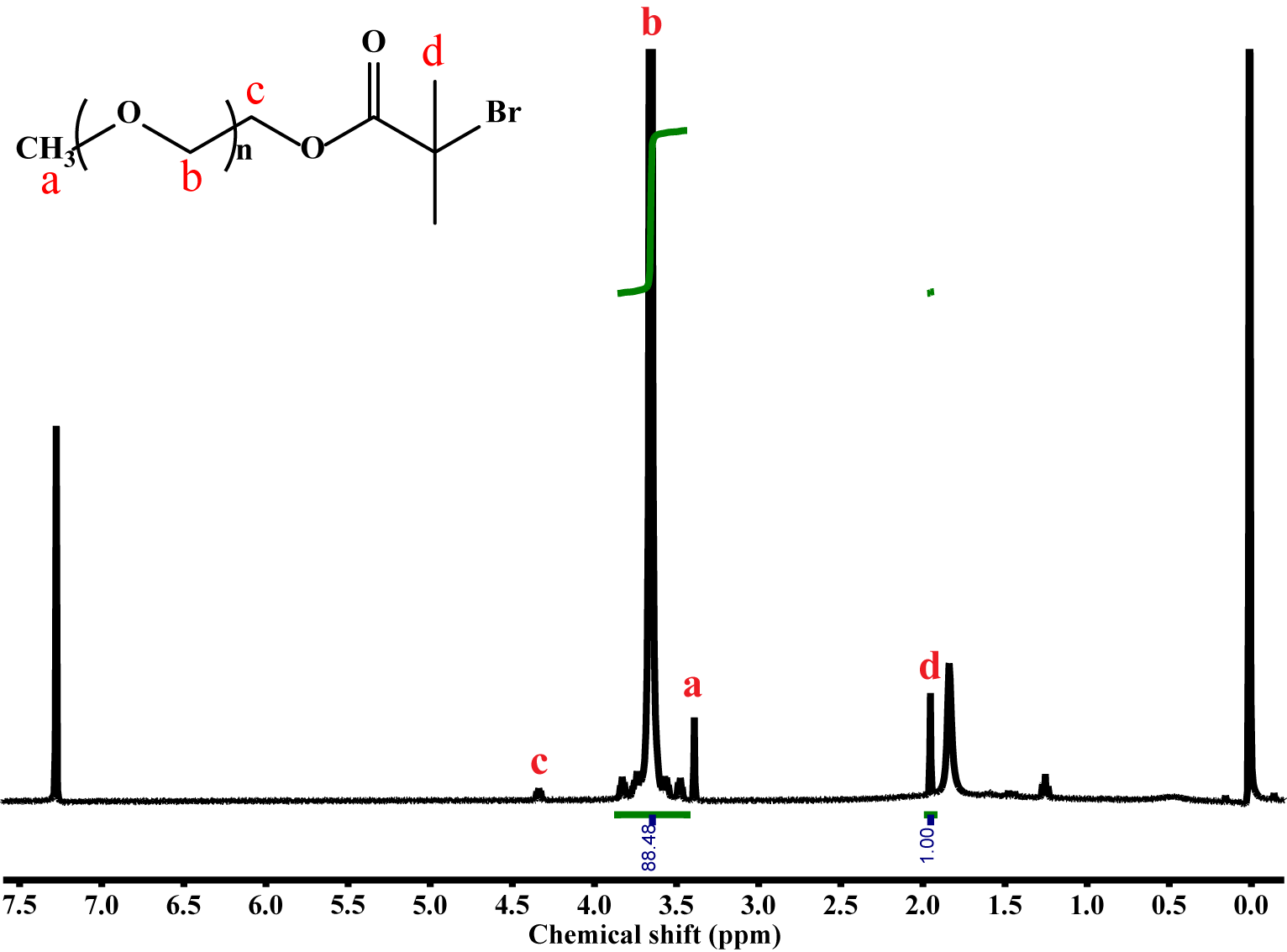


**Figure S1.** ^1^H-NMR spectrum of the MePEG-Br macroinitiator.


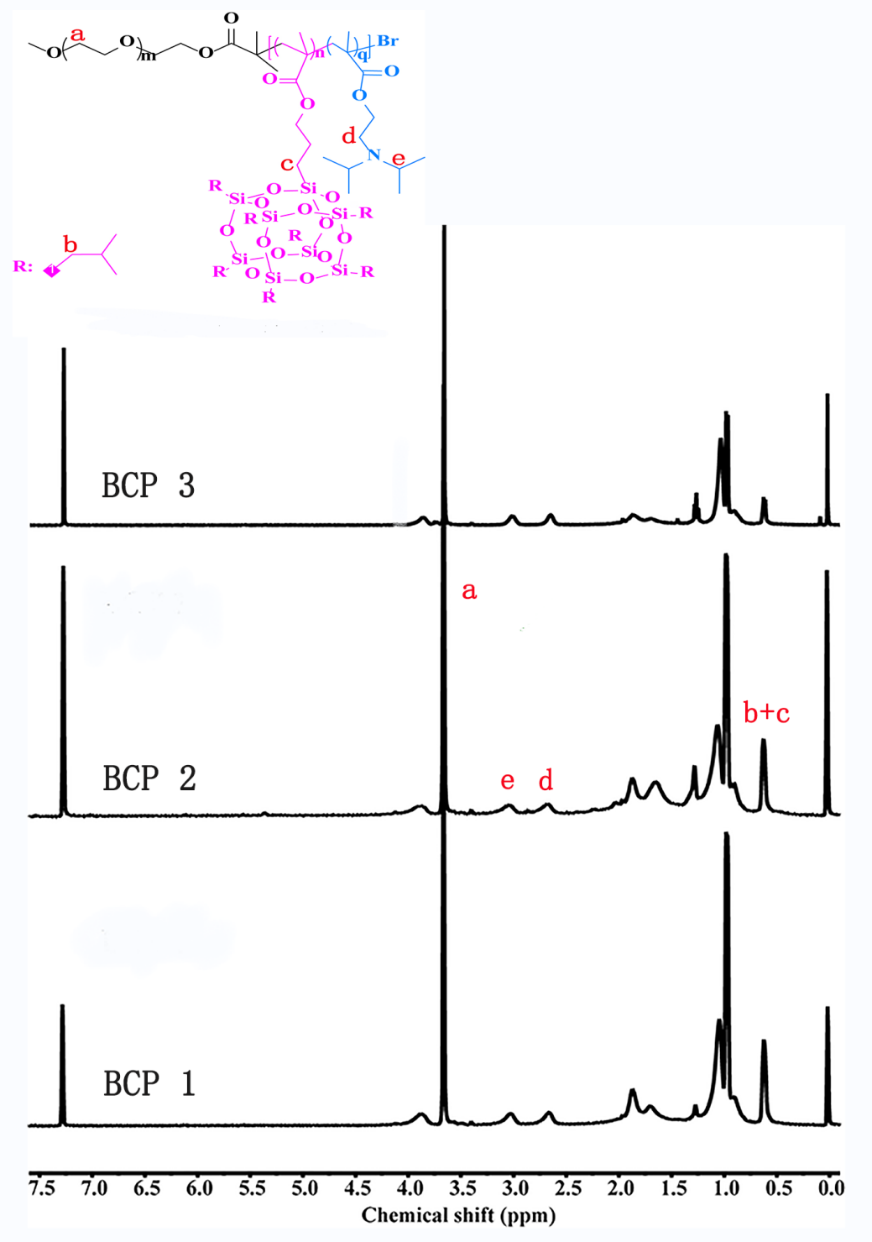


**Figure S2.** ^1^H-NMR spectra of MePEG-*b*-P(MAPOSS-*co*-DPA) with various molecular weights.


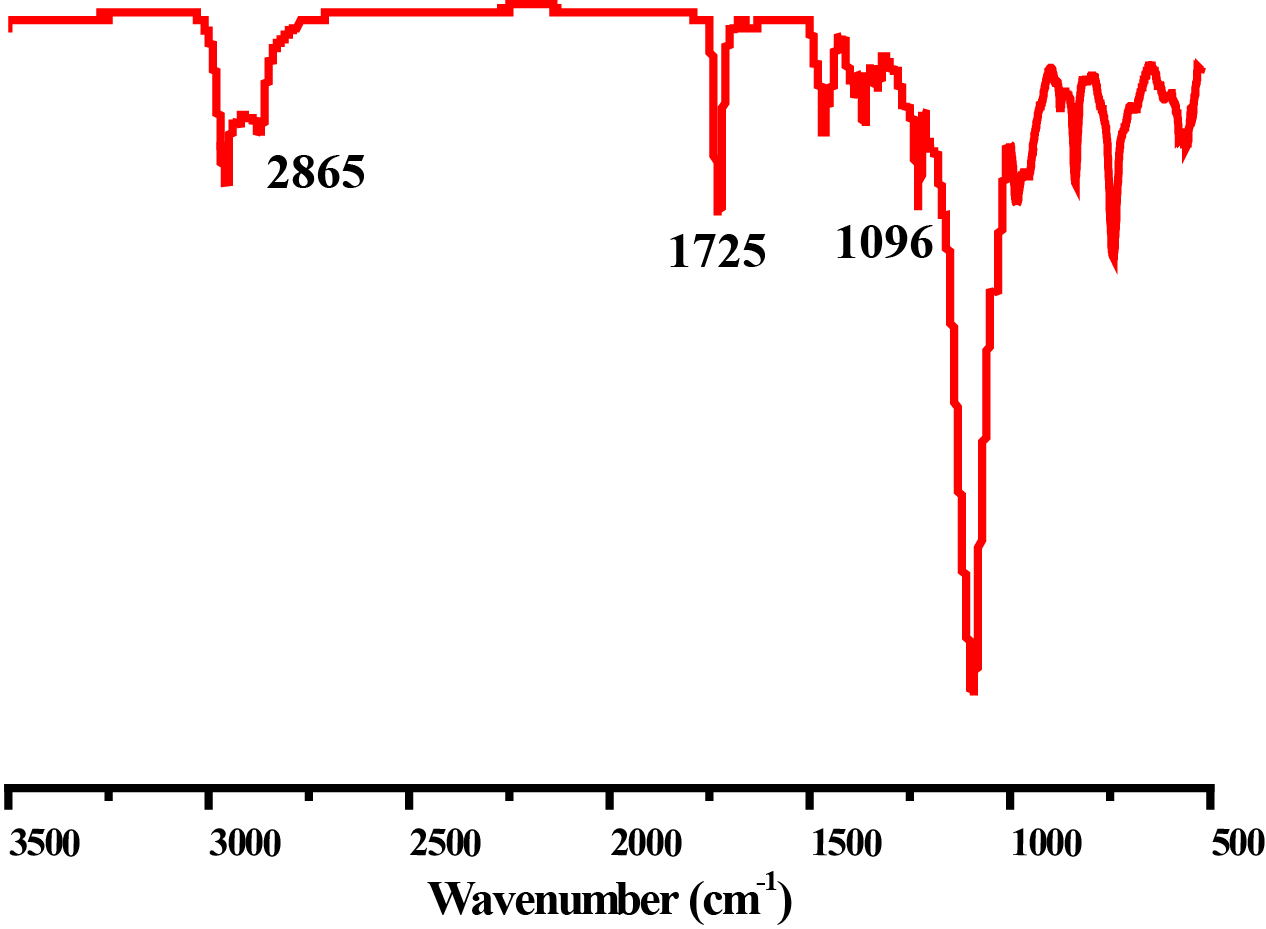


**Figure S3.** FTIR spectrum of MePEG-*b*-P (MAPOSS-*co*-DPA).


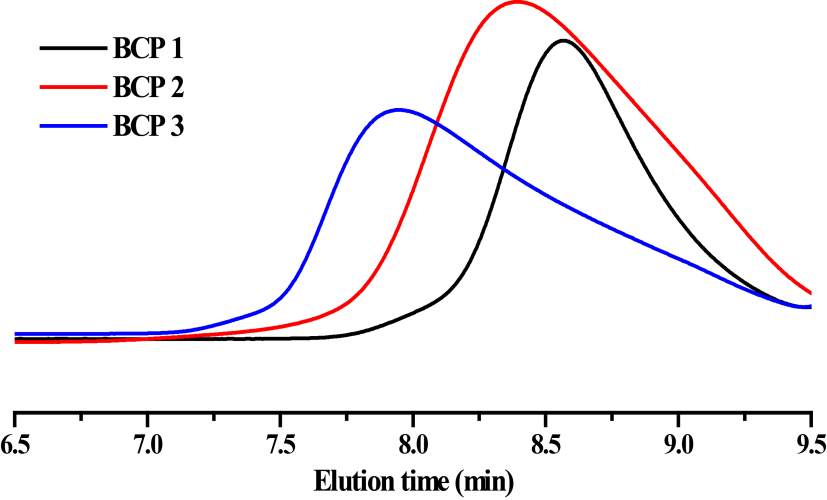


**Figure S4.** GPC traces of MePEG-*b*-P(MAPOSS-*co*-DPA).


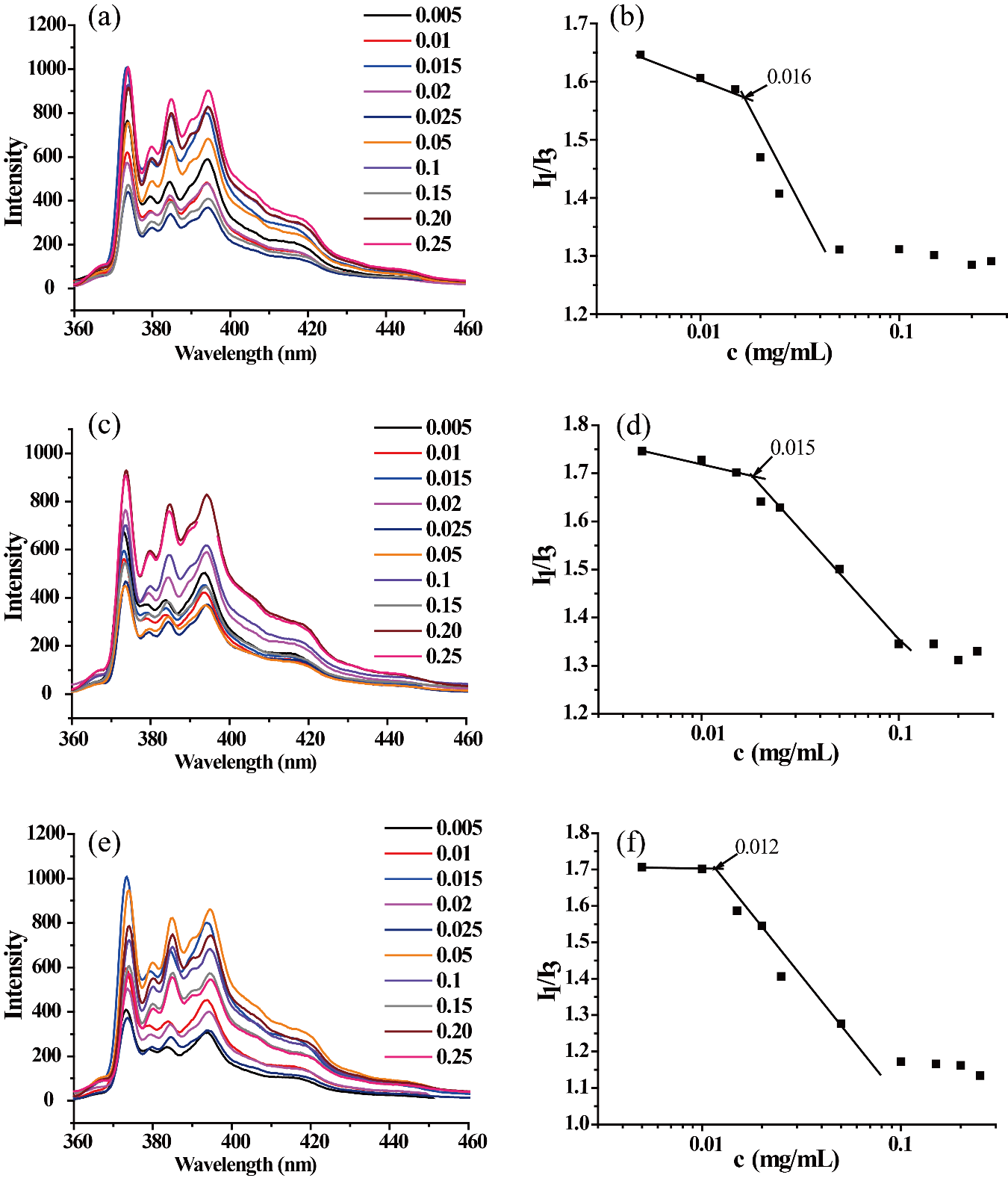


**Figure S5.** Fluorescence-emission spectrogram of pyrene in an aqueous solution of amphiphilic block copolymer: (**a**) BCP1; (**c**) BCP2 and (**e**) BCP3 with different concentrations (mg/mL); relationship between *I*_1_/*I*_3_ of pyrene and amphiphilic block copolymer concentration: (**b**) BCP1, (**d**) BCP2 and (**f**) BCP3.


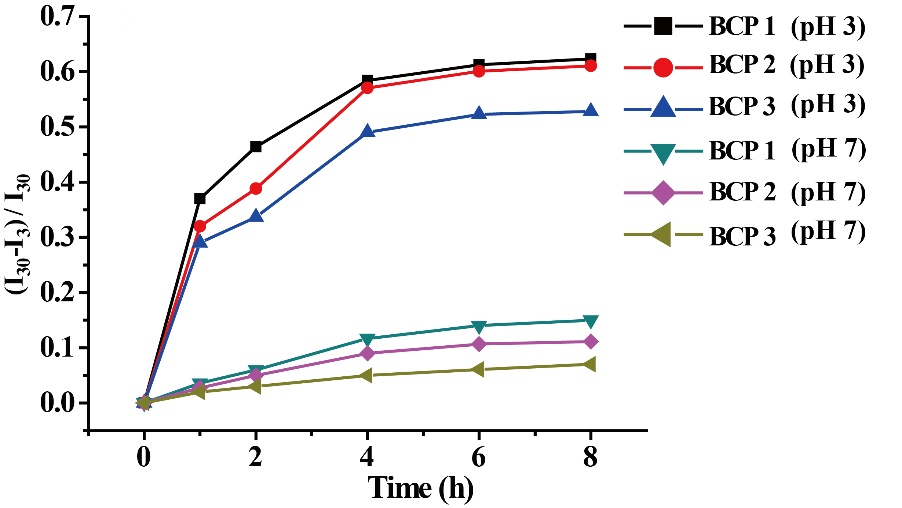


**Figure S6.** Cumulative pyrene release percentage of pyrene encapsulated by micelles of BCP1, BCP2 and BCP3 when pH = 7 and pH = 3.
